# A whirl of radiomics-based biomarkers in cancer immunotherapy, why is large scale validation still lacking?

**DOI:** 10.1038/s41698-024-00534-9

**Published:** 2024-02-21

**Authors:** Marta Ligero, Bente Gielen, Victor Navarro, Pablo Cresta Morgado, Olivia Prior, Rodrigo Dienstmann, Paolo Nuciforo, Stefano Trebeschi, Regina Beets-Tan, Evis Sala, Elena Garralda, Raquel Perez-Lopez

**Affiliations:** 1 Radiomics Group, Vall d’Hebron Institute of Oncology (VHIO), Barcelona, Spain; 2 Oncology Data Science (ODysSey) Group, Vall d’Hebron Institute of Oncology (VHIO), Barcelona, Spain; 3 Department of Medical Oncology, Vall d’Hebron University Hospital and Institute of Oncology (VHIO), Barcelona, Spain; 4 Prostate Cancer Translational Research Group, Institute of Oncology (VHIO), Vall d’Hebron University Hospital, Barcelona, Spain; 5 Molecular Oncology Group, Vall d’Hebron University Hospital and Institute of Oncology (VHIO), Barcelona, Spain; 6 Department of Radiology, Netherlands Cancer Institute, Amsterdam, The Netherlands; 7 GROW School for Oncology and Reproduction, Maastricht University, Maastricht, The Netherlands; 8 Faculty of Health Sciences, University of Southern Denmark, Odense, Denmark; 9 Dipartimento Diagnostica per Immagini, Radioterapia Oncologica ed Ematologia, Policlinico Universitario A. Gemelli IRCCS, Rome, Italy; 10 Dipartimento di Scienze Radiologiche ed Ematologiche, Universita Cattolica del Sacro Cuore, Rome, Italy

**Keywords:** Cancer imaging, Predictive markers

## Abstract

The search for understanding immunotherapy response has sparked interest in diverse areas of oncology, with artificial intelligence (AI) and radiomics emerging as promising tools, capable of gathering large amounts of information to identify suitable patients for treatment. The application of AI in radiology has grown, driven by the hypothesis that radiology images capture tumor phenotypes and thus could provide valuable insights into immunotherapy response likelihood. However, despite the rapid growth of studies, no algorithms in the field have reached clinical implementation, mainly due to the lack of standardized methods, hampering study comparisons and reproducibility across different datasets. In this review, we performed a comprehensive assessment of published data to identify sources of variability in radiomics study design that hinder the comparison of the different model performance and, therefore, clinical implementation. Subsequently, we conducted a use-case meta-analysis using homogenous studies to assess the overall performance of radiomics in estimating programmed death-ligand 1 (PD-L1) expression. Our findings indicate that, despite numerous attempts to predict immunotherapy response, only a limited number of studies share comparable methodologies and report sufficient data about cohorts and methods to be suitable for meta-analysis. Nevertheless, although only a few studies meet these criteria, their promising results underscore the importance of ongoing standardization and benchmarking efforts. This review highlights the importance of uniformity in study design and reporting. Such standardization is crucial to enable meaningful comparisons and demonstrate the validity of biomarkers across diverse populations, facilitating their implementation into the immunotherapy patient selection process.

## Introduction

Cancer immunotherapy, particularly immune checkpoint inhibitors (ICI), has emerged as the gold standard for treating various cancers, including lung, renal, and melanoma^1–4^. The remarkable success achieved with ICI has generated optimism for its potential application in treating numerous other types of cancer. However, the variability in patient responses makes it necessary to identify biomarkers capable of predicting individual responses to ICI. This crucial step is instrumental in enhancing patient stratification, maximizing treatment efficacy, detecting treatment resistance and thus minimizing potential harm for those who may not benefit. Various tissue-based predictive biomarkers have been proposed, such as microsatellite instability (MSI)^5,6^, tumor mutational burden (TMB)^7^, programmed death-ligand 1 (PD-L1) expression^8^, and tumor-infiltrating lymphocyte (TIL) count^9^. However, these biomarkers often require invasive procedures to obtain tumor tissue for analysis, and their accuracy in identifying suitable candidates for immunotherapy remains suboptimal^10^. Radiomics analysis, in combination with machine learning (ML) methods, efficiently extracts meaningful information from medical images, enabling three-dimensional evaluation of tumors throughout the entire body, and repeated assessments over the course of cancer treatment^11^. In particular, extracting radiomics features from standard-of-care CT images, a widely used imaging technique for cancer staging and follow-up, offers a valuable tool with potential for developing predictive biomarkers in the context of immunotherapy^12–15^. This is especially pertinent in cancer immunotherapy, where treatment may occur after the initial diagnosis, in pretreated patients with evolving tumors and non-reachable lesions^16,17^. The non-invasive nature of radiomics applications thus becomes highly valuable.

In fact, the emergence of encouraging radiomics signatures for predicting response to immunotherapy has caused a boom in research endeavors in this field. Nevertheless, the absence of standardized protocols and benchmarking studies of biological validation of such signatures poses a significant challenge for the application of these signatures in clinical practice. Despite numerous radiomics studies predicting response across various tumor types, inconsistencies persist in data selection, model construction, and outcome definition. To assess the reliability of predictive radiomics studies, standardization research criteria such as the Radiomics Quality Score (RQS)^11^ and the CLEAR checklist^18^ have been introduced^19^. However, low RQS have been reported in most published radiomics studies, indicating poor documentation practices and limited reproducibility^20^. Efforts are emerging to develop PRISMA-AI guidelines^21^ that will define standardized frameworks, comprehensive method descriptions, and data-sharing practices in radiomics-based studies, as well as, allow study comparison, validation, and meta-analysis efforts in this domain.

In this review, we provide an overview of the current state of radiomics-based biomarkers to guide the use of immunotherapy through a comprehensive examination. It encompasses the potential biases and variations in the currently developed radiomics pipelines that challenge the comparison of studies through meta-analysis. Additionally, we present a short case study featuring a meta-analysis of studies predicting PD-L1 status from CT imaging, comparing radiomics ML and deep learning (DL) models. By examining the existing literature and conducting a meta-analysis, we aim to offer valuable insights and perspectives on the efficacy and reliability of radiomics as immunotherapy biomarkers.

## Results

### Uncovering potential sources of variability in radiomics study design

**Fig. 1:**
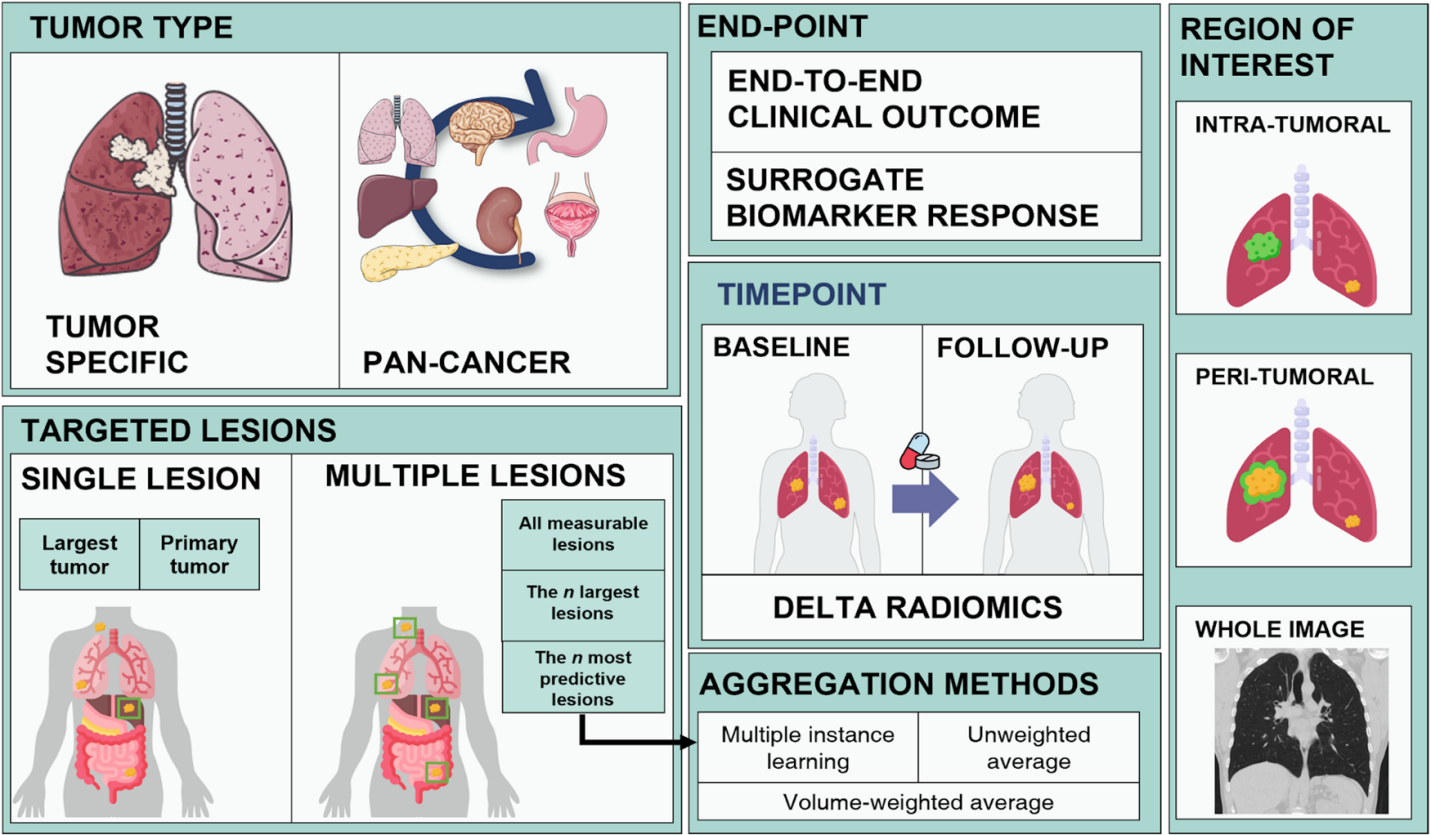
Potential sources of variability in radiomics study design including features related to the cohort setting (specific signature for a single tumor type or pan-cancer), end-point (for clinical outcome such as response yes/no or for predicting molecular surrogate biomarkers such as programmed death-ligand 1 [PD-L1] expression), number of lesions, imaging timepoints and region of interest. *n: number of lesions*.

We conducted a systematic review encompassing all studies utilizing ML or DL techniques in CT imaging for predicting either direct response to immunotherapy or any surrogate biomarker of response. Our findings highlight the significant diversity in study design among publications aiming to create similar predictive models (Fig. 1). This variability in methodology presents a challenge when attempting to compare the performance of these models through meta-analysis. In this section, we aim to summarize all these studies and the differences among them.

#### Cohort setting

The characteristics that define the tumor phenotype and make it more responsive to immunotherapy can encompass tumor-specific aspects or those that can be expressed and captured across multiple tumor types. Consequently, researchers have pursued two approaches in the development and validation of radiomics-based biomarkers. One approach being tumor specific, aiming to create validated biomarkers within each type. While the other approach addresses this challenge by incorporating multiple tumor types and considering the location of metastatic disease when feeding the models.

To date, the majority of radiomics studies on immunotherapy response prediction have focused on non-small cell lung cancer (NSCLC), benefiting from the availability of larger datasets and a higher degree of treatment responses in this tumor type. In fact, most of the studies exploring radiomics to predict direct response or surrogates of response to immunotherapy (i.e., biological and molecular markers used as predictors of a patient’s response such as PD-L1) have been done in NSCLC populations. Other tumor types including melanoma, gastric, head and neck, bladder and kidney cancers have been investigated to a lesser extent and only few studies have developed predictive radiomics models in pan-cancer settings^12,14^. Despite the increasing number of lung cancer and melanoma patients receiving immunotherapy as part of standard care, it is noteworthy that only around 30% of the articles included cohorts larger than 200 patients, and merely 22% reported the utilization of external validation cohorts (Supplementary Table 1).

The development of tumor type-specific radiomics signatures allows finding radiomics features unique to that population; however, it reduces the generalization of the methods to other tumor types that are less common or rarely treated with immunotherapy. On the other hand, pan-cancer approaches require the use of larger cohorts for the model to comprehend the inherent heterogeneity of the population, thereby reducing the bias towards the response probability of each tumor type.

#### Outcome evaluation

Studies focusing on predictive radiomics signatures and immunotherapy can be categorized into two types: those aiming to directly predict clinical outcome and those focused on predicting known surrogate biomarkers. However, the lack of standardization regarding outcome definition poses a significant challenge, making it difficult to compare and assess the predictive capabilities of the resulting radiomics signatures.

One major challenge is the wide range of clinical endpoints used to assess treatment response. The most relevant measure for evaluating the benefit of immunotherapy treatment in patients is overall survival (OS). While certain radiomics studies have considered OS as the clinical endpoint^13,15,22–33^, most studies rely on tumor size changes by the Response Evaluation Criteria in Solid Tumors version 1.1 (RECIST 1.1)^16^. From the RECIST assessment, multiple measurements can be computed and used as endpoints, including progression-free survival (PFS)^29–32,34–38^, disease control (which gathers complete response (CR), partial response (PR), and stable disease (SD))^14,22,28,34,35,39–46^ or objective response rate (ORR)^23,47–50^. However, it is important to note that these response evaluations are considered surrogate endpoints for OS, and their reliability is hindered by their inherent subjectivity and variability, challenging the development of reproducible models^51,52^. Furthermore, the wide range of response evaluation criteria derived from RECIST^53,54^ (e.g., PFS, ORR, disease control) also limits the direct comparison of radiomics signatures across studies.

Similarly, when predicting molecular surrogate biomarkers (such as PD-L1 expression), many studies tend to discretize the target variable and transform it into a classification problem. However, these biomarker cutoffs are subjected to the primary tumor biology or the type of treatment. Therefore, the lack of standardized cutoff values further complicates the comparison of radiomics signatures for predicting surrogate biomarkers in immunotherapy. In addition to the heterogeneity in endpoint definitions, it is important to consider that the performance of radiomics signatures predicting surrogate biomarkers will be inherently limited by the predictive capacity of the surrogate biomarker itself. This implies that the effectiveness of the radiomics signatures in predicting treatment response will be constrained by the predictive capabilities of the surrogate biomarker being used.

#### Study design regarding number of lesions, region of interest and time-points

Another relevant point in the study design for immunotherapy radiomics signatures is the selection of target lesions for analysis. Many radiomics studies rely on delineating and extracting features from a single selected tumor (~63% of the studies found in the review), often the primary or largest lesion, arguing that the single chosen lesion can represent the whole disease. However, in patients with metastases at multiple sites, heterogeneous immunophenotypes can drive different immune responses^55,56^. Therefore, analyzing only one lesion per patient may not fully capture the tumor heterogeneity and limit the predictive capacity of the model. To partially overcome this limitation, feature aggregation methods such as average, volume-weighted average, or attention-based multiple instance learning (MIL) are commonly used^14,41,49,57^. Additionally, the analysis of inter- and intra-lesion heterogeneity through radiomics studies to capture the whole metastatic disease has also been considered as a potential indicator of immunotherapy response^58^.

Moreover, with the aim of providing the model with all the potential relevant data and knowing the effect of surrounding tumor microenvironment for immunotherapy response, certain studies have also explored the value of incorporating peritumoral area information into predictive models for predicting response to immunotherapy^22,50^. Nevertheless, the models obtained more relevant information from the intratumoral features. Some studies have also shown that intratumoral 3D radiomics features provide more informative insights compared to using only 2D radiomics features^28^.

Finally, regarding the imaging time-points, the majority of studies in radiomics research have focused on the development of predictive biomarkers using baseline scans, which refer to the scans obtained just before initiating treatment. This approach facilitates improved patient selection for treatment decision-making. However, some studies have demonstrated enhanced outcomes by analyzing changes in the radiomics tumor phenotype between baseline and early follow-up time points, commonly known as early readouts or delta radiomics signatures^13,15,22,29,43,47,59^. Such approaches enable the capture of response or progression patterns that may go unnoticed by radiologists, thereby potentially preventing patients to stay longer under ineffective treatment. Some of these studies have shown that tracking these changes in CT scans provides better prognostic value compared to the current standard of care, RECIST^13,60^. It is important to note, however, that these early readouts do not represent true predictive biomarkers per se, but rather serve as indicators of early response, and should be thought of as alternative response criteria themselves, rather than predictive biomarkers. This is because at the time these early readouts are assessed, treatment decisions have already been made, and the patient is already receiving immunotherapy.

#### Radiomics feature selection and model implementation

Fifty percent of the pipelines implemented for hand-crafted radiomics analysis correspond to Least Absolute Shrinkage and Selection Operator (LASSO) for feature selection (implemented in 40% of the studies), followed by a logistic regression for classification (implemented in 25% of the studies). Multiple studies have highlighted the benefits of utilizing LASSO as the feature selection method due to its efficacy in high-dimensional data regression, thereby mitigating the risk of overfitting^30,49^. In terms of classification method, several studies have explored the performance of different classification algorithms for predicting response (such as support vector machine (SVM), Random Forest (RF), decision tree and k-nearest neighbor) (Supplementary Figure 1). All of them showed that logistic regression had similar or slightly better performance than other more complex classifiers^28,38,50,61^.

Only a few studies have used more advanced DL methods to predict response to immunotherapy^13,32,45,46^. These methods are data-hungry and need large cohorts of patients, as well as reliable and objective annotations, to achieve good performance. However, gathering this amount of data regarding immunotherapy treatment response is still challenging. For that reason, most of the CT-based DL models currently developed are focused on predicting surrogates of response such as PD-L1 status^32,62–64^.

### Case-study: meta-analysis for predicting PD-L1 status from CT imaging comparing DL vs classical ML

**Fig. 2:**
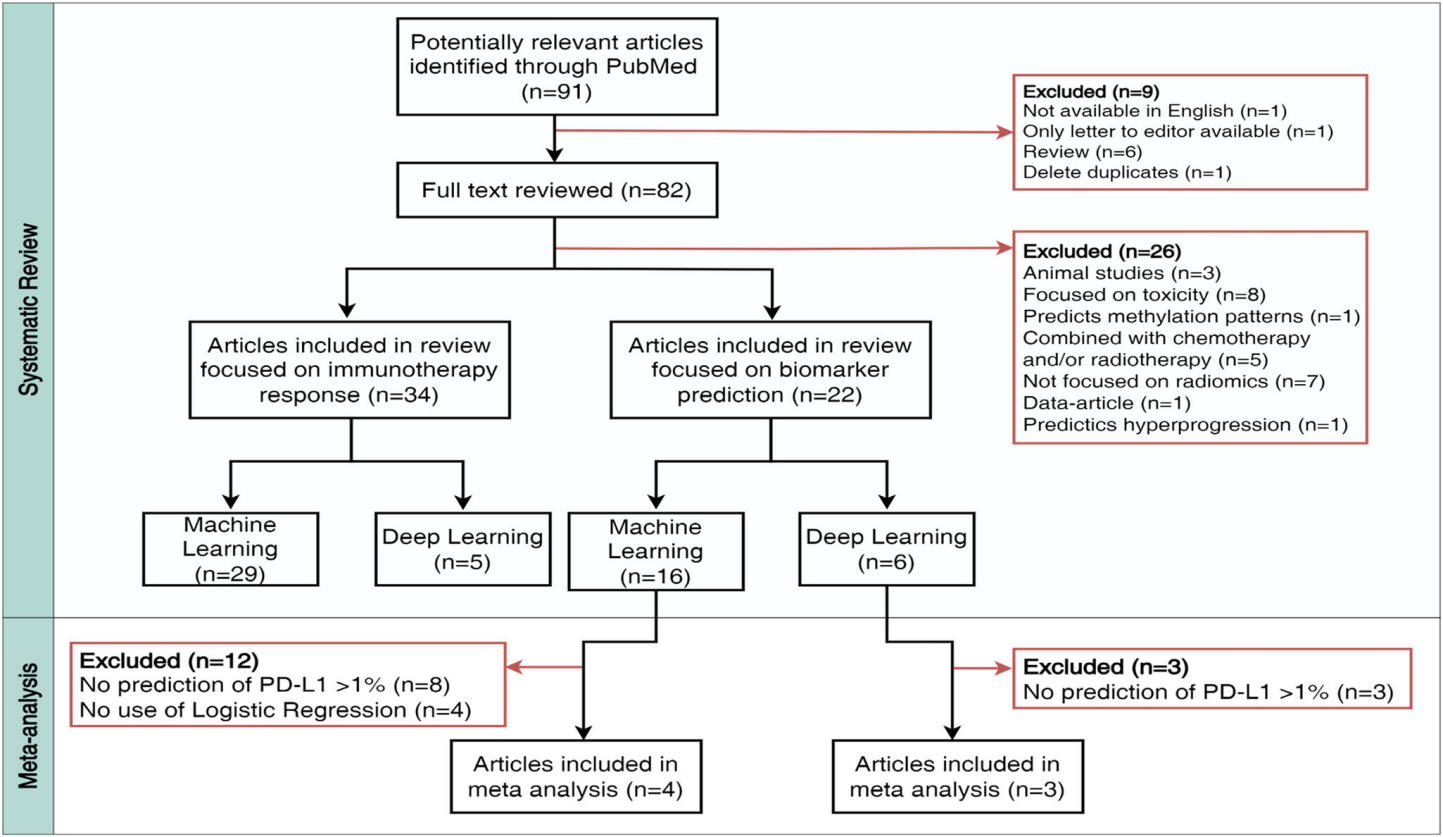
Referred Reporting Items for Systematic Reviews and Meta-Analyses (PRISMA) flow diagram for study illustrating the number of records screened in the review and articles included and excluded, outlining the predefined inclusion and exclusion criteria. In total, 56 articles were included in the review and 35 articles were excluded, reasons for exclusion were reported. Seven studies exploring CT-based radiomics models for predicting programmed death-ligand 1 (PD-L1) expression were included in the meta-analysis.

In order to get a better understanding of the overall performance of the radiomics signatures as predicting biomarkers for immunotherapy, we conducted a meta-analysis of all the studies that implemented CT-based radiomics with classical ML or DL to predict PD-L1 status. Figure 2 shows a flow chart illustrating the systematic review conducted in PubMed, outlining the predefined inclusion and exclusion criteria.

We identified a total of 56 articles developing CT-based predictive signatures in patients treated with immunotherapy; 34 for predicting direct response and 22 predicting surrogate molecular biomarkers (Supplementary. Detailed results systematic review). In Supplementary Table 1, all included papers are listed. We reviewed the CLEAR guidelines for all these studies (Supplementary Table 2). However, we could not include harmonized image preprocessing techniques or feature selection methods. Accounting for the previously described variability in the methods of radiomics signatures and with the aim of investigating the most standardized models, we found seven comparable studies to perform the meta-analysis. All of them predicted PD-L1 expression assessed as tumor proportion score (TPS) ≥ 1%, using the area under the curve (AUC) as the evaluation metric and implementing either logistic regression (n = 4)^61,64–66^ or DL (n = 3)^62,64,67^ as the predictive model. External validation performance was also explored in three studies applying logistic regression methods and one DL modeling.

**Fig. 3:**
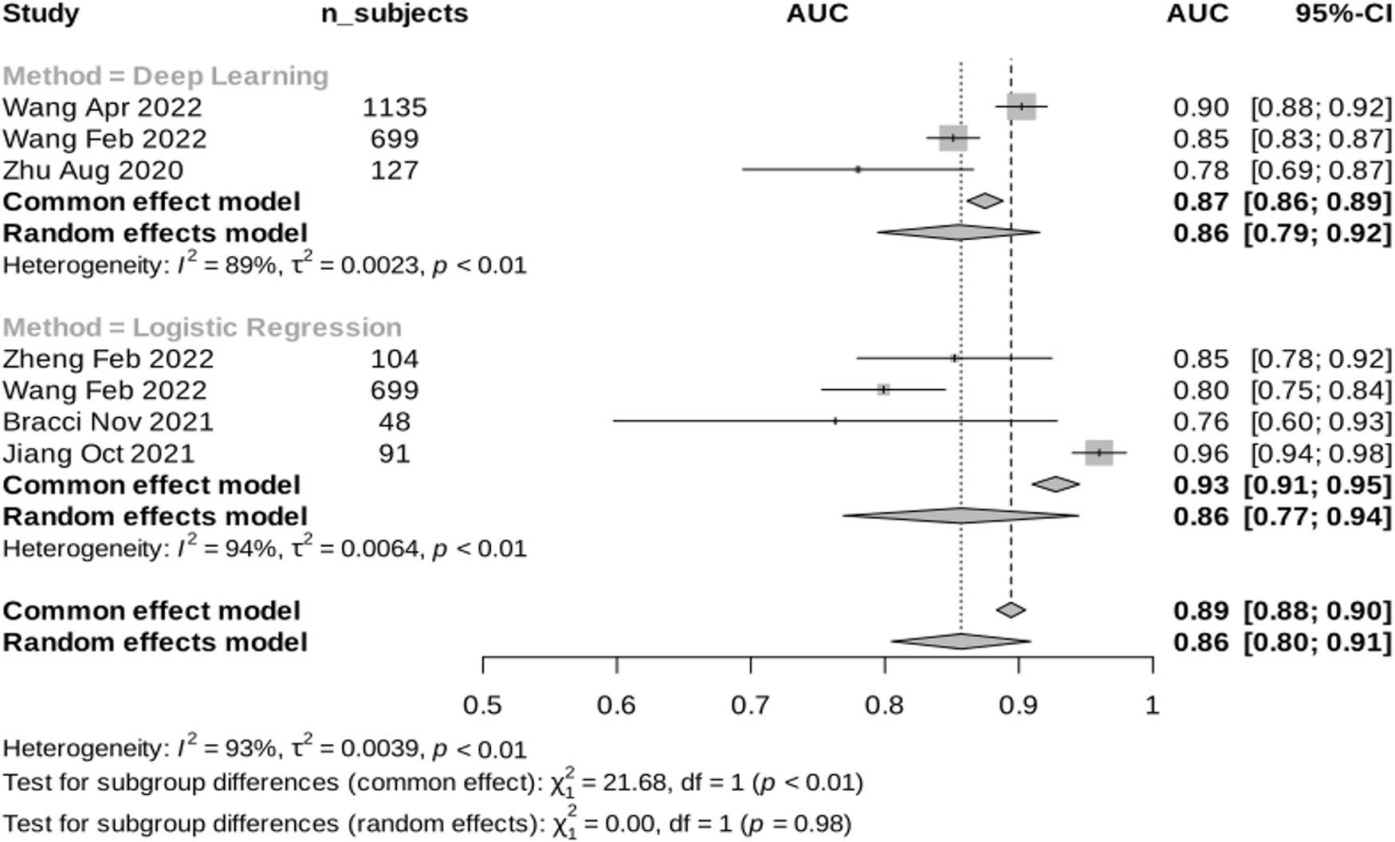
Meta-analysis results: Internal validation performance of the reported studies that implemented CT-based radiomics with classical machine learning (ML) or deep learning (DL) for predicting programmed death-ligand 1 (PD-L1) expression.

**Fig. 4:**
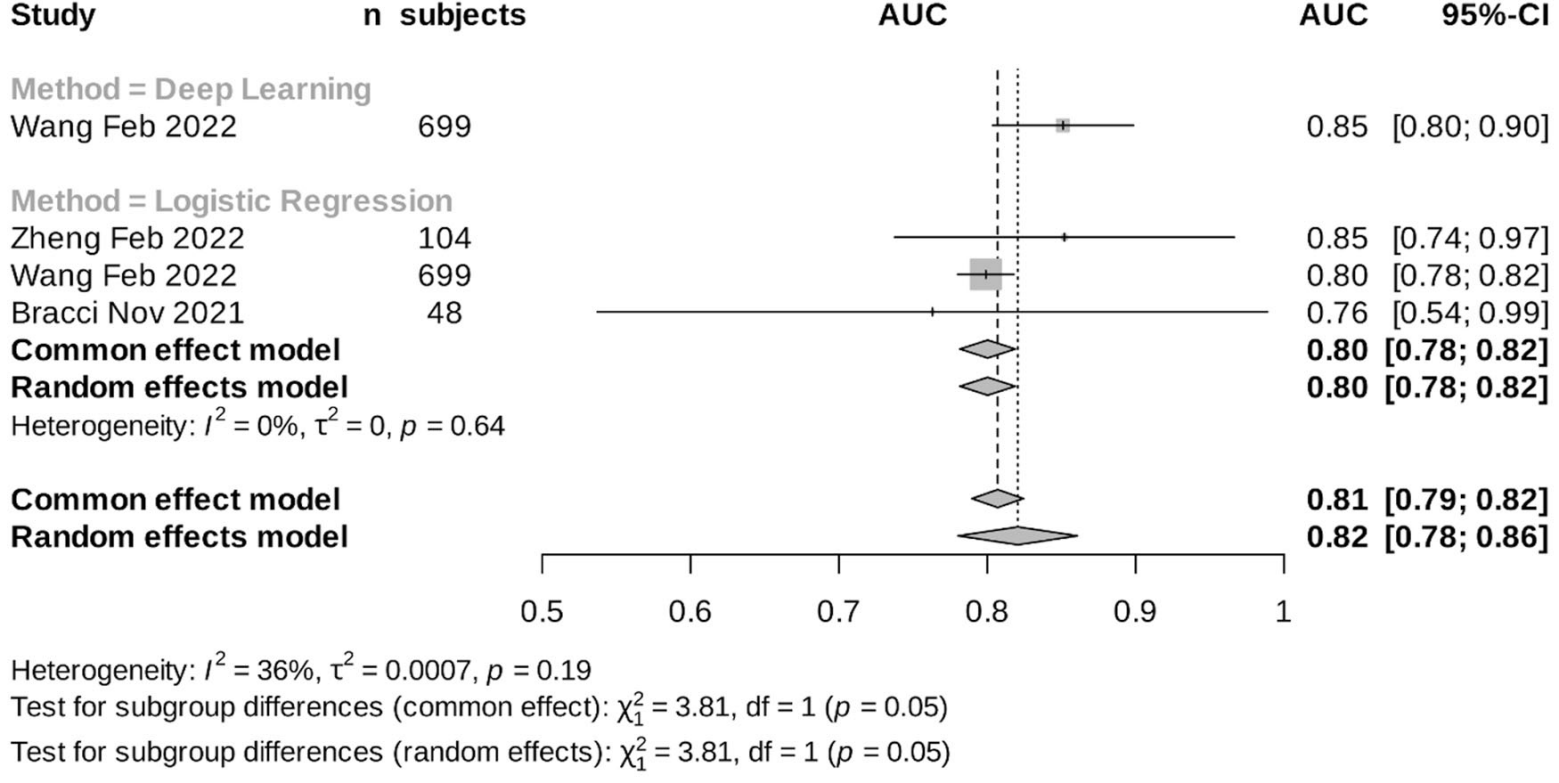
Meta-analysis results: External validation performance of the reported studies that implemented CT-based radiomics with machine learning (ML) or deep learning (DL) for predicting programmed death-ligand 1 (PD-L1) expression.

The included papers showed varying performance in predicting PD-L1 expression, with AUROCs ranging from 0.76 to 0.96 in both logistic regression and DL methods. In the internal validation, the logistic regression models showed a pooled AUC of 0.86 (95%CI 0.77–0.94, i²= 94%) while the DL method exhibited a pooled AUROC of 0.86 (95%CI 0.79–0.92, i²= 89%), using random effects model (Fig. 3). Interestingly, our findings revealed that the performance across different studies for logistic regression remained comparable in the external validation set, yielding an estimated AUROC of 0.80 (95%CI 0.78–0.82, i²= 0%) (Fig. 4). These findings indicate low heterogeneity between studies in the external validation performance in contrast to the higher heterogeneity in the internal set. There was not enough data from DL studies to evaluate the heterogeneity in the external set. Notably, studies utilizing logistic regression and DL methods demonstrated similar results in the internal set, with a combined estimated AUROC of 0.86 (95% CI 0.80–0.91), despite DL models having access to a larger dataset compared to logistic regression studies.

## Discussion

The application of artificial intelligence (AI) to improve patient stratification towards better treatment selection is of growing interest in both radiology and oncology fields. Numerous studies have focused on uncovering radiological features of tumors that could predict response patterns to immunotherapy. However, the lack of standardized and homogeneous frameworks employed in these studies, as well as scarce data sharing, present challenges when comparing results and validating radiomics models, ultimately, hindering their integration into clinical practice. In this review, we aimed to shed light on the factors that contribute to the variability in radiomics studies, rendering the available models incomparable. Additionally, we conducted a comprehensive literature review specifically targeting studies that investigated CT-based radiomics signatures for predicting response to immune checkpoint inhibitors (ICI) and surrogate biomarkers of response to ICI.

Remarkably, our findings revealed that despite the abundance of studies predicting direct response to immunotherapy, only a limited number of these studies employed similar methods, making them unsuitable for meta-analysis. This significant variability in methodology poses challenges in terms of study comparisons and reproducibility across different datasets. Similar challenges have arisen in the development of other potential predictive biomarkers based on biological samples such as PD-L1 expression or TMB, highlighted in debates surrounding the heterogeneous distribution of these markers in tumor samples, variations in staining techniques, and establishment of appropriate thresholds, among other issues^68–70^. Addressing these challenges requires benchmarking studies that facilitate the comparison of established methods with novel techniques across diverse cohorts, thereby promoting advancement and standardization in the field.

Nonetheless, some studies investigating the development of radiomics signatures for predicting programmed death-ligand 1 (PD-L1) expression in tumors met the necessary criteria for meaningful pooling and meta-analysis. Consequently, our meta-analysis exclusively focused on studies examining CT-based radiomics for predicting PD-L1 status. This case study highlighted the promising performance of the reported models in predicting PD-L1 expression, with area under the receiver operating characteristic curve (AUROC) values ranging from 0.7 to 0.9. While these models have demonstrated positive outcomes and exhibited limited heterogeneity in accuracy during external validation, questions persist regarding the lack of widespread adoption in clinical practice. One potential contributing factor could be the absence of a reported correlation between PD-L1 prediction and treatment response. Furthermore, it is important to acknowledge the potential influence of publication bias, which may result in a prevalence of positive results, possibly overshadowing scientifically crucial findings from studies that may not achieve high accuracy despite employing sound methodologies.

Developing multi-center studies is essential to demonstrate the applicability of these methods across large and heterogeneous datasets, ensuring reliability and fairness by encompassing diverse populations and machines from various institutions. Concerns regarding data privacy and patient data monetization have slowed down the development of large-scale multi-center models. Nevertheless, efforts have been made in this field to provide more secure methods of data sharing and decentralized model training, such as federated learning^71,72^, where models can be trained on multi-institutional data without leaving the respective institutions, thus safeguarding data privacy. Moreover, some studies have highlighted potential improvements of predictive models through multimodal approaches that combine radiomics with histopathology or genomics^73,74^. Still, this requires representative heterogeneous data ideally from multiple centers, including all sources of information, which has been a notable limitation thus far. Finally, integration of radiomics-based biomarkers into clinical practice hinges on the critical aspects of explainability and trustability, ensuring that healthcare professionals can comprehend and rely on these complex data-driven insights to make informed patient care decisions.

Moreover, the path to integrating radiomics into clinical practice, even when all the previous limitations are considered, still relies on biological translation of the predictive models. Certain studies have made substantial progress in this direction by correlating radiomics predictions with biological and molecular markers like PD-L1^30^, cellular pathways^39^ or cytotoxic immunophenotype^14^. Other studies have focused on developing models that aim to predict directly the molecular properties of the tumor from surgical resections or biopsies^75^. However, ongoing investigation in this direction is needed to enhance the reliability and applicability of these models for seamless integration into routine clinical practice.

In conclusion, the journey towards establishing radiomics-based biomarkers is challenging, requiring technical development of imaging assays and computational methods, validation encompassing sensitivity, specificity, and reproducibility evaluations, biological validation, as well as proving clinical relevance ideally through embedding them in prospective clinical trials. Despite the considerable interest and expectations from the scientific community, as well as the abundance of papers exploring imaging phenotypes derived from radiomics as potential biomarkers of response to immunotherapy, these tools have yet to be implemented in clinical practice. To make a substantial impact on clinical trials and medical practice, larger prospective studies with appropriate external validation datasets, focusing on the clinical applicability of these signatures, are crucial.

Fortunately, changes are underway in the field that should facilitate the exploration of these novel biomarkers and their potential applicability in the clinic. The imaging scientific community, through collaborative efforts and consortia supported by the EU commissioner, is working to bridge the gap between research and real-world application. Among the most significant initiatives is the EUCAIM project, which is dedicated to establish an infrastructure for over 60 million cancer images from over 100,000 cancer patients with the goal to develop and benchmark trustworthy AI tools. Together, we strive to pave the way for the true integration of radiomics-based biomarkers into clinical decision-making, ultimately improving the care of cancer patients.

## Methods

### Detailed description of the of the systematic review methodology

#### Search strategy

A search was conducted in the PubMed electronic database for potential articles published at date October 1st, 2022. The search strategy used was *(((“Radiomics” OR “CT based biomarker” OR “imaging based biomarker” OR “imaging marker” OR “imaging biomarker”) AND (“Immunotherapy”[Mesh] OR “ipilimumab” OR “tremelimumab” OR “CTLA-4” OR pembrolizumab” OR nivolumab” OR “Immuno Checkpoint Inhibitors”[Mesh] OR “cemiplimab” OR “atezolizumab” OR “immune checkpoint blockade” OR “avelumab” OR “durvalumab” OR “PD-L1” OR “PD-1”)) AND (((“Tomography, X-Ray Computed” [Mesh] OR “Computed Tomography” OR “CT”) NOT “Positron Emission Tomography”) NOT “PET”)*. Our search terms did not include specific cancer types or outcome types. Finally, we also considered any articles referred to us by experts, identified during the prior scoping search, or found in the references section of the full-text articles we evaluated.

Instead of only assessing studies based on hand-crafted radiomics applied to classical machine learning (ML) models, studies that employed deep learning (DL) techniques were also examined. Articles were evaluated systematically on title and full-text level, and reasons for exclusion were noted. All studies which were potentially relevant for the paper were included in a data extraction table.

#### Study selection and eligibility criteria

According to the inclusion criteria, we focused exclusively on systemic treatments involving immune checkpoint inhibitors (ICI) alone. Articles were included if they were (i) primary studies that investigated (ii) response to ICIs alone by using (iii) classical ML or DL on (iv) human tumor lesions and (v) written in the English language.

We excluded studies of ICI in combination with other therapies. If the study included patients who received immunotherapy, chemotherapy and/or radiotherapy, we only included them in case the results for immunotherapy were assessed separately. Other forms of immunotherapy, such as monoclonal antibodies, vaccines, immune system modulators, or T-cell transfer therapy, were beyond the scope of our review. Predicting hyperprogression, toxicity and methylation patterns were also considered outside the scope of this review.

The included articles were divided in two different categories, based on the type of predicted outcome; prediction of end-to-end ICI response or biomarkers for response. Then, for every outcome category, we divided the studies based on the applied methods: conventional ML and DL approaches. From each article, we reported the used methods, main results and the reported conclusions and limitations. Regarding the methods, we collected the feature aggregation and selection, and the implemented ML algorithm. We filtered the results from some studies with additional experiments regarding other endpoints, as defined in the exclusion criteria.

#### Statistical analysis

To obtain an overall estimation, the area under the curve (AUC) with 95% confidence interval (CI) was calculated for each study. No p-values were reported for pooled AUCs. Heterogeneity estimation was assessed and reported in all analyses using means of I2 and a statistical test to evaluate the similarity of results across studies (homogeneity test). Both fixed and random effects models were applied regardless of the homogeneity test outcome. When the p-value was greater than 0.05 (indicating no significant heterogeneity), the fixed effects model was used, assuming a common effect size. Conversely, the random effects model, employing the DerSimonian-Laird method, was utilized to account for heterogeneity. Due to limited statistical power in detecting heterogeneity, the random effects model was employed for subgroup analysis.

Internal validation results, accounting for cross-validation and internal split, were used for the meta-analysis. External validation was also analyzed when applicable in an additional experiment. All the analyses were implemented using R v(4.2.2) and package metafor.

### Supplementary information

Supplementary
