# Supplementary material for: A whirl of radiomics-based biomarkers in cancer immunotherapy, why is large scale validation still lacking?
Source: NPJ Precis Oncol. 2024 Feb 21;8:42. doi: 10.1038/s41698-024-00534-9 (PMC10881558; doi:10.1038/s41698-024-00534-9)

Supplementary Notes

Detailed results of the systematic review

The used search query resulted in a total of 91 articles. The selection process is shown in Figure 2. Nine studies were excluded as they were non-primary research, not available in English or duplicates. After reviewing the full text, 26 studies were excluded. Studies were excluded as they involved non-human subjects, predicted an end-point that was not included in the criteria, combined different therapies or did not focus on radiomics. In total, 56 studies are included in the review.

Supplementary Table 1. Description of all the published papers of CT-based radiomics signatures for predicting response to immunotherapy or surrogate biomarkers using either machine learning (ML) or deep learning (DL) methods. *Abbreviations: NSCLC (Non-small Cell Lung Cancer), OS (Overall Survival), PFS (Progression Free Survival).*

Study	Primary cancer	Total cohort	End point(s)
**Machine Learning for Response Prediction**			
Trebeschi S et al. 2019 Jun	Melanoma<br>NSCLC	203	Disease control
Ligero M et al. 2021 Apr	Solid Tumors	198	Disease control
Liu Y et al. 2021 Mar	NSCLC	197	Disease control
Wu M et al. 2022 Jan	NSCLC	131	Disease control
Ji Z et al. 2020 Oct	Gastric	87	Disease control
Colen R et al. 2021 Apr	Rare cancers	57	Disease control
Wang Z et al. 2020 Aug	Melanoma	50	Disease control
Malone E et al. 2022 Feb	Renall Cell Carcinoma	27	Disease control
Khorrami M et al. 2020 Jan	NSCLC	139	Disease control, OS
Yang Y et al. 2021 Feb	NSCLC	308	Disease control, PFS
Yang B et al. 2021 Jun	NSCLC	92	Disease control, PFS
Gong J et al. 2022 Jan	NSCLC	224	Objective response
Liang Z et al. 2022 Jan	Gastric	87	Objective response
Park K et al. 2020 Oct	Urothelial carcinoma	62	Objective response
Yuan G et al. 2021 Jan	Hepatocellular carcinoma	58	Objective response
Peisen F et al. 2022 Jun	Melanoma	262	Objective response, OS
Tunali I et al. 2021 May	NSCLC	332	OS
Schraag A et al. 2019 Dec	Melanoma	103	OS
Corino V et al. 2021 May	Head and Neck	85	OS
Zerunian M et al. 2021 Mar	NSCLC	21	OS
Dercle L et al. 2022 Mar	Melanoma	575	OS
Ungan G et al. 2022 Oct	Melanoma	71	OS, Disease control
Guerrisi A et al. 2021 Oct	Melanoma	32	OS, PFS
Dercle L et al. 2020 May	NSCLC	92	PFS
Ladwa R et al. 2020 Jul	NSCLC	47	PFS
Liu C et al. 2021 Feb	NSCLC	46	PFS
Jazieh K et al. 2022 Mar	NSCLC	133	PFS, OS
Nardone V et al. 2020 Feb	NSCLC	59	PFS, OS
Tunali I et al. 2019 Mar	NSCLC	228	TTP

**Deep Learning for Response Prediction**			
Ren Q et al. 2022 Aug	NSCLC	157	Disease control
Rundo F et al. 2021 Oct	Urothelial carcinoma	42	Disease control
Trebeschi S et al. 2021 Mar	NSCLC	152	OS
He B et al. 2022 Apr	NSCLC	236	OS, PFS
Trebeschi S et al. 2021 Apr	Urothelial	74	OS
**Machine Learning for Biomarker Prediction**			
Sun R et al. 2018 Sep	Solid Tumors	491	CD8
Varghese B et al. 2022 Sep	Renall Cell Carcinoma	78	CD8-T, PD-L1
Huang W et al. 2022 Aug	Gastric	2272	NLR
Mazzaschi G et al. 2020 Jun	NSCLC	100	OS
Trentini F et al. 2022 Feb	NSCLC	100	PD-1/CD8 ratio
Tang C et al. 2018 Jan	NSCLC	290	PD-L1
Zheng Y et al. 2022 Jan	Head and Neck carcinoma	179	PD-L1
Zheng Y et al. 2022 Aug	Head and Neck carcinoma	157	PD-L1
Yoon J et al. 2020 Apr	Lung	153	PD-L1
Iwatate Y et al. 2020 Oct	Pancreatic	107	PD-L1
Bracci S et al. 2021 Nov	NSCLC	72	PD-L1
Shiinoki T et al. 2022 Feb	NSCLC	161	PD-L1
Jiang Z et al. 2021 Oct	NSCLC	125	PD-L1
Wen Q et al. 2021 Aug	NSCLC	120	PD-L1
Wen Q et al. 2020 Nov	Esophageal Squamous	220	PD-L1, CD8
Tang X et al. 2021 Jul	Bladder	75	TMB
**Deep Learning for Biomarker Prediction**			
Wang C et al. 2021 Dec	NSCLC	1262	EGFR Mutation, PD-L1
Wang C et al. 2022 Feb	NSCLC	873	EGFR Mutation, PD-L1
Wang C et al. 2022 Apr	NSCLC	1946	PD-L1
Tian P et al. 2021 Jan	NSCLC	939	PD-L1
Zhu Y et al. 2020 Aug	Lung	127	PD-L1
He B et al. 2020 Jul	NSCLC	327	TMB

Supplementary Table 2. CLEAR guidelines applied to the seven articles included in the meta-analysis.

Title	Wang Feb 2022	Wang Apr 2022	Zhu Aug 2020	Zheng Feb 2022	Wang Feb 2022	Bracci Nov 2021	Jiang Oct 2021
1 Relevant title, specifying the radiomic methodology	NO	YES	NO	YES	NO	YES	YES
**Abstract**							
2 Structured summary with relevant information	Yes	YES	YES	YES	Yes	YES	YES
**Keywords**							
3 Relevant keywords for radiomics	NO	YES	YES	YES	NO	YES	YES
**Introduction**							
4 Scientific or clinical background	YES	YES	YES	YES	YES	YES	YES
5 Rationale for using a radiomic approach	YES	YES	YES	YES	YES	YES	YES
6 Study objective(s)	YES	YES	YES	YES	YES	YES	YES
**Method**							
7 Adherence to guidelines or checklists (e.g., CLEAR checklist)	NO	NO	YES	NO	NO	NO	NO
8 Ethical details (e.g., approval, consent, data protection)	YES	YES	YES	NO	YES	YES	YES
9 Sample size calculation	NO	NO	NO	NO	NO	NO	NO
10 Study nature (e.g., retrospective, prospective)	YES	YES	NO	NO	YES	YES	YES
11 Eligibility criteria	YES	YES	NO	YES	YES	YES	YES
12 Flowchart for technical pipeline	YES	YES	NO	NO	YES	NO	NO
13 Data source (e.g., private, public)	*YES*	YES	YES	YES	*YES*	*YES*	YES
14 Data overlap	NA	NA	NA	NA	NA	NA	NA
15 Data split methodology	YES	YES	YES	NO	YES	YES	YES

16 Imaging protocol (i.e., image acquisition and processing)	YES	NO	YES	YES	YES	YES	YES
17 Definition of non-radiomic predictor variables	YES	YES	YES	NO	YES	NO	NA
18 Definition of the reference standard (i.e., outcome variable)	YES	YES	YES	YES	YES	YES	YES
19 Segmentation strategy	*YES*	*NO*	YES	*YES*	*YES*	*YES*	*YES*
20 Details of operators performing segmentation	NO	NO	YES	YES	NO	YES	YES
21 Image pre-processing details	*YES*	YES	YES	YES	*YES*	*NO*	*YES*
22 Resampling method and its parameters	YES	YES	NO	YES	YES	NO	YES
23 Discretization method and its parameters	YES	YES	YES	YES	YES	NO	YES
24 Image types (e.g., original, filtered, transformed)	YES	NO	NO	NO	YES	NO	NO
25 Feature extraction method	NO	YES	*NA*	YES	NO	*YES*	*YES*
26 Feature classes	YES	YES	NA	YES	YES	YES	YES
27 Number of features	YES	YES	NA	YES	YES	YES	YES
28 Default configuration statement for remaining parameters	NO	YES	NA	YES	NO	NO	NO
29 Handling of missing data	*NA*	*NA*	*NA*	*NA*	*NA*	*NA*	*NA*
30 Details of class imbalance	NO	YES	NO	NA	NO	YES	YES
31 Details of segmentation reliability analysis	NO	NO	NO	YES	NO	YES	YES
32 Feature scaling details (e.g., normalization, standardization)	NO	NO	NO	YES	NO	NO	NO
33 Dimension reduction details	YES	YES	NA	YES	YES	YES	YES
34 Algorithm details	YES	YES	YES	YES	YES	YES	YES
35 Training and tuning details	NO	YES	YES	YES	NO	YES	YES
36 Handling of confounders	NO	NO	NO	NO	NO	NO	NO
37 Model selection strategy	NO	YES	NO	YES	NO	NO	YES

38 Testing technique (e.g., internal, external)	YES	YES	YES	YES	YES	YES	YES
39 Performance metrics and rationale for choosing	YES	YES	YES	YES	YES	YES	YES
40 Uncertainty evaluation and measures (e.g., confidence intervals)	YES	YES	NO	YES	YES	YES	NO
41 Statistical performance comparison (e.g., DeLong's test)	YES	YES	YES	YES	YES	YES	YES
42 Comparison with non-radiomic and combined methods	YES	YES	NO	NO	YES	NO	YES
43 Interpretability and explainability methods	YES	YES	no	NO	YES	NO	NO
**Results**							
44 Baseline demographic and clinical characteristics	YES	YES	YES	YES	YES	YES	YES
45 Flowchart for eligibility criteria	YES	YES	NO	YES	YES	NO	YES
46 Feature statistics (e.g., reproducibility, feature selection)	NO	NO	NA	YES	NO	NO	NO
47 Model performance evaluation	YES	YES	YES	YES	YES	YES	YES
48 Comparison with non-radiomic and combined approaches	YES	YES	NO	NO	YES	YES	YES
**Discussion**							
49 Overview of important findings	YES	NO	YES	YES	YES	YES	YES
50 Previous works with differences from the current study	NO	YES	YES	YES	NO	YES	NO
51 Practical implications	YES	YES	YES	YES	YES	YES	YES
52 Strengths and limitations (e.g., bias and generalizability issues)	YES	YES	YES	YES	YES	YES	YES
**Open Science**							
53 Sharing images along with segmentation data [n/e]	NO	NO	NO	NO	NO	NO	NO
54 Sharing radiomic feature data	NO	NO	NO	NO	NO	NO	NO
55 Sharing pre-processing scripts or settings	NO	NO	NO	NO	NO	NO	NO
56 Sharing source code for modeling	NO	NO	NO	NO	NO	NO	NO
57 Sharing final model files	NO	NO	NO	NO	NO	NO	NO
58 Sharing a ready-to-use system [n/e]	NO	NO	NO	NO	NO	NO	NO

Supplementary Figure 1. Most common feature selection and modeling methods. Graph showing the most frequently used feature selection methods and machine learning models (ML) in combination for predicting direct response or surrogate biomarkers of response. 'Other' includes the less frequent methods used. *Abbreviations: LASSO (least absolute shrinkage and selection operator), mRMR (minimal-redundancy-maximal-relevance), SVM (support vector machine), LogReg (Logistic Regression).*

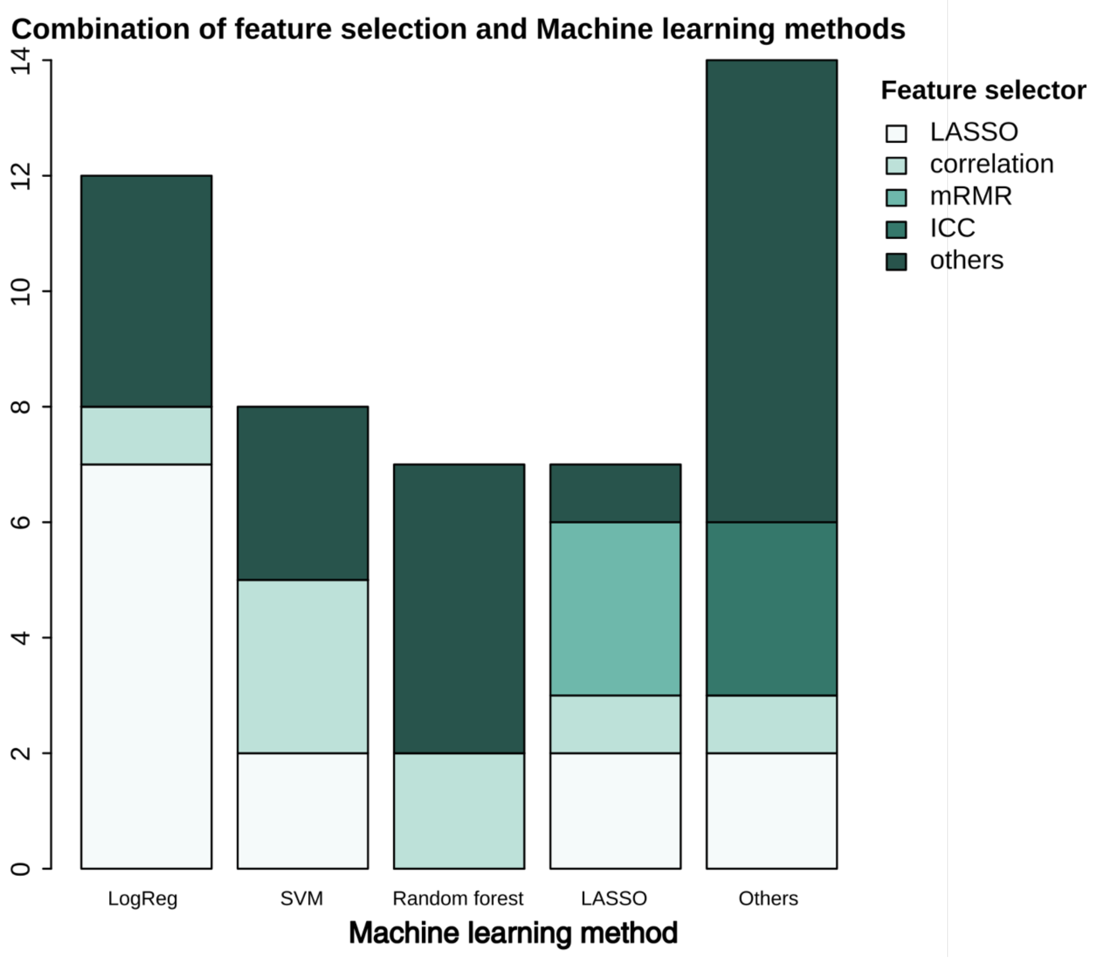

Supplement: Supplementary file 1 — Supplementary [file 41698_2024_534_MOESM1_ESM.pdf]
